# Acknowledgement to Reviewers of *Toxics* in 2014

**DOI:** 10.3390/toxics3010018

**Published:** 2015-01-08

**Authors:** Toxics Editorial Office

**Affiliations:** MDPI AG, Klybeckstrasse 64, CH-4057 Basel, Switzerland

The editors of *Toxics* would like to express their sincere gratitude to the following reviewers for assessing manuscripts in 2014:

Alvarado, Cynthia G.
Angelini, Sabrina
Bai, Xiaowen
Barmpas, Fotios
Batley, Graeme
Beevers, Sean
Bernhoft, Robin
Biasioli, Franco
Bielmyer, G.K.
Bouris, Dimitris
Braue, Jr, Ernest H.
Brinkmann, Markus
Brosseau, Lisa M
Bushnell, Tim
Büsselberg, Dietrich
Bustamante, Paco
Carls, Mark G.
Colosio, Claudio
Cory-Slechta, Deborah A.
Crisponi, Guido
Cristol, Daniel
de Groot, Didima
Díaz-Cruz, M. Silvia
Ferrante, Margherita
Forbes, Valery E.
Franklin, Jade
Frederiksen, Hanne
Fritsche, Ellen
Geier, Mark R.
George, Graham
González, M. J.
Henryk, Kozlowski
Heussen, Henri
Hider, Robert C.
Horrillo, María del Carmen
Irato, Paola
Isani, Gloria
Kakosimos, Konstantinos
Kalinich, John
Kanda, Isao
Kanungo, Jyotshna
Klanjscek, Tin
Kodavanti, Prasada Rao S.
Koskela, Harri T.
Kozlowski, Henryk
Kvítek, Libor
Lebrun, Jérémie D.
Li, Yanli
Linkov, Igor
Lucchini, Roberto
Mahmoudi, Morteza
Mari, Montse
Massarsky, Andrey
Mikler, John
Mori, Kouki
Morita, Kyoji
Nawrot, Tim
Nott, David M.
Oliveira Fernandes, José
Padilla, Stephanie
Petroianu, Georg A.
Peyton, Brent M
Pitschmann, Vladimir
Qin, Xiaosheng
Raushel, Frank M.
Robinson, Stacey A.
San Jose, Roberto
Scheringer, Martin
Seto, Yasuo
Shafer, Timothy J.
Simcik, Matt F.
impraga, M.
Sinicropi, Maria Stefania
Smith, Philip
Souidi, Maamar
Spada, Lucia
Spisni, Enzo
Suzuki, Yayoi
Tamás, Markus J.
Thèvenod, Frank
Thiermann, H.
Tierney, Keith B.
Torreblanca, Amparo
Usenko, Crystal
Valcárcel, Y.
Vallero, Daniel A.
van Thriel, Christoph
Verlicchi, Paola
Vighi, Marco
Vilavert, Lolita
Volberg, Vitaly
von Goetz, Natalie
Warren, Joshua
Yang, Albert C.
Yoshida, Kunihiro
Zarros, Apostolos

